# Nephrogenic Syndrome of Inappropriate Antidiuresis

**DOI:** 10.1155/2012/937175

**Published:** 2012-02-28

**Authors:** D. Morin, J. Tenenbaum, B. Ranchin, T. Durroux

**Affiliations:** ^1^Centre de Référence des Maladies Rénales Rares du Sud-Ouest, Néphrologie Pédiatrique, CHU Montpellier, Université Montpellier 1, 34295 Montpellier, France; ^2^CNRS, UMR 5203, Inserm U661, IGF, Université Montpellier 1-2, 34094 Montpellier, France; ^3^Service de Néphrologie Pédiatrique, Centre de Référence des Maladies Rénales Rares, Hôpital Femme-Mère-Enfant, Hospices Civils de Lyon, UFR de Médecine Lyon-Est et Inserm U499, Université Claude Bernard Lyon I, 69500 Lyon, France

## Abstract

Mutations in the vasopressin V2 receptor gene are responsible for two human tubular disorders: X-linked congenital nephrogenic diabetes insipidus, due to a loss of function of the mutant V2 receptor, and the nephrogenic syndrome of inappropriate antidiuresis, due to a constitutive activation of the mutant V2 receptor. This latter recently described disease may be diagnosed from infancy to adulthood, as some carriers remain asymptomatic for many years. Symptomatic children, however, typically present with clinical and biological features suggesting inappropriate antidiuretic hormone secretion with severe hyponatremia and high urine osmolality, but a low plasma arginine vasopressin level. To date, only two missense mutations in the vasopressin V2 receptor gene have been found in the reported patients. The pathophysiology of the disease requires fuller elucidation as the phenotypic variability observed in patients bearing the same mutations remains unexplained. The treatment is mainly preventive with fluid restriction, but urea may also be proposed.

## 1. Introduction

Since 1992, when the vasopressin V2 receptor gene (*AVPR2*) sequence that codes for the V2 receptor (V2R) was first described [1], more than 200 mutations in the *AVPR2* have been found in patients presenting with X-linked congenital nephrogenic diabetes insipidus (cNDI) [2, 3]. Functional studies of these mutant receptors have demonstrated a loss of function in the mutated protein that results in the insensitivity of the renal collecting duct to the action of the arginine vasopressin hormone (AVP). This in turn leads to a defect in water reabsorption with polyuria, polydipsia, and hypernatremic dehydration.

Recently, it was demonstrated that the V2R may be affected by gain of function mutations that cause a new syndrome: the nephrogenic syndrome of inappropriate antidiuresis (NSIAD) [4]. This phenomenon has been observed for other G-protein-coupled receptors, for example, TSH (thyrotoxicosis), LH (familial male-limited precocious puberty), PTH-rp (Bloom syndrome), and the calcium sensing receptors (hypercalciuric hypocalcemia). NSIAD was subsequently described in two infant boys presenting with seizures due to severe hyponatremia and high urinary osmolality, but low plasma AVP levels. *AVPR2 *sequencing demonstrated that these two children harbored the arginine-137-cysteine (R137C) and arginine-137-leucine (R137L) mutations in their respective V2 receptors. Since 2005, when the disease was first described, all the NSAID patients presented in the literature have had one of these two *AVPR2 *mutations [4–10]. Functional studies have shown that both mutations are responsible for a constitutive activation of the mutant V2R, leading to inadequate water reabsorption in spite of low AVP levels [4, 11]. Nevertheless, the clinical presentations of these NSIAD patients have been highly variable, with one of them showing severe neurological consequences, while others were fully asymptomatic with only a urine dilution defect revealed during water-load testing [5, 6]. Thus, patients may be diagnosed early in life or in adulthood, or they may remain asymptomatic. The diagnosis of NSIAD thus should be systematically considered in cases of childhood hyponatremia, especially when associated with high urine osmolality.

## 2. Pathophysiology of NSIAD

AVP is synthesized in the supraoptic and paraventricular nuclei and acts through three types of AVP receptors: the V1a receptor (vasopressive effects of AVP), the V1b receptor found in the adenohypophysis (ACTH secretion), and the V2 receptor (V2R) localized in the distal renal collecting duct [12]. At the cellular level, AVP-V2R binding initiates a cascade of events, with adenosine 3 : 5-cyclic phosphate (cAMP) production through stimulated V2R coupling with the G*α*s protein and the activation of adenylyl cyclase. Intracytoplasmic protein phosphorylation by cAMP-dependent protein kinase A therefore occurs, which leads to the exocytic insertion of aquaporin 2 (AQP2), a specific water channel, into the luminal membrane of the principal cells of the renal collecting duct, thereby increasing its water permeability.

Under normal conditions, AVP activation of the V2R also leads to phosphorylation of serine residues located in the C-terminal receptor tail with, secondarily, *β*-arrestin recruitment and V2R internalization [13]. This negative regulation of the V2R after stimulation by AVP prevents prolonged and excessive tubular reabsorption of water. In NSIAD, the constitutively active mutant V2R appears to lose this important property, at least partly, but by a mechanism that remains not fully understood.

Functional studies of the R137C- and R137L-V2R mutants have shown that both mutants have increased basal cAMP production compared with the Wt-V2R, confirming their constitutive activity [4, 11]. It has also been shown that their relatively low amplitude constitutive activities are only weakly sensitive to the action of a V2R-inverse agonist like the SR121463 compound [11]. These data suggest that these mutant receptors are mainly in an almost blocked conformation and are consistent with Decaux's observation that NSIAD patients are insensitive to nonpeptide V2R inverse agonists [6]. Nevertheless, it has also been shown that the relatively low AVP-induced stimulation of both mutants is completely inhibited by V2R inverse agonists. Therefore, this latter finding suggests that, although these mutant receptors are more often in a blocked conformation, they can also adopt a more flexible conformation capable of a desensitization/internalization process [11].

## 3. Clinical Presentation

All patients diagnosed with NSIAD to date have been boys and most diagnoses were made during the first two years of life. Most patients appeared to have a relatively clear phenotype with biological features initially suggesting a syndrome of inappropriate antidiuretic hormone secretion (SIADH) with hyponatremia, low serum osmolality, and unexpectedly high urine osmolality, but low plasma AVP levels. The main clinical features, seizure or irritability, appeared to be linked to the severe hyponatremia.

During the neonatal period, boys bearing the mutated receptor seem able to enough dilute their urine and therefore avoid the risk of severe hyponatremia. Indeed, unless peculiar clinical conditions such as neonatal asphyxia, the physiological limitation in concentration capacity during the first weeks of life may protect them from excessive water reabsorption during this period.

Two large kindreds of patients with NSIAD have also been reported. Decaux et al. reported the first kindred and showed that the male patients in this family had been diagnosed in adulthood and were doing well, suggesting that NSIAD conditions may sometimes remain unrecognized until advanced age [6]. The late diagnosis in such patients may also be explained by the drop in water excretion naturally observed in the elderly [14].

The female carriers of this X-linked disorder were also clinically asymptomatic, but some of them demonstrated an impaired ability to dilute urine during a water-load test performed with 20 mL/kg of water.

More recently, we reported another four-generation family with NSIAD bearing the R137C-V2R mutation [5]. In this family, the clinical presentation was highly variable in the male carriers. Indeed, one of the children had two episodes of seizures related to hyponatremia with low plasma AVP level, when he was 10 months and 34 months. Both episodes occurred during the summertime when large amounts of water were given to this young boy to prevent dehydration. His older brother, also bearing the mutated V2R, lived in the same city and, as far as we know, had the same diet but remained fully asymptomatic. Unfortunately, a first cousin, also having the same *AVPR2 *mutation, experienced several seizure episodes due to recurrent hyponatremia between 27 months and 5 years of age and eventually developed permanent mental retardation.

A water-load test was performed in the asymptomatic male carrier (10 mL/kg water load) and the heterozygous females (20 mL/kg water load). The results showed that water loading in the hemizygous male carrier was characterized by persistent AQP2 urine excretion independently of concomitant vasopressin excretion. This finding is consistent with a constitutive activation of the V2R. Slow and incomplete water elimination with slight hyponatremia was also found. The female carriers displayed variable biological findings after water loading that can be explained by random X inactivation [5].

Among all the family members explored, only the symptomatic hemizygous male exhibited all the expected signs under basal conditions: persistent AQP2 urine excretion, low output of highly concentrated urine, and low plasma and urine AVP levels. His asymptomatic brother, bearing the same mutation, had low AQP2 excretion and normal urine output under basal conditions but displayed a total absence of downregulation of AQP2 excretion after a cautious 10 mL/kg water load.

Concerning the plasma AVP levels measured in these NSIAD patients, most levels appeared to be low or undetectable in spite of hyponatremia and high urine osmolality. Nevertheless, in at least one case, it was shown that AVP production can persist despite low plasma sodium and plasma osmolality [8]. Thus, of the four SIADH categories described, NSIAD most often belongs to the D group with a low plasma AVP levels, although it can also be grouped in category B, which is characterized by a measurable plasma AVP level [8, 15]. The explanation for this persistent AVP secretion found in some patients despite low plasma osmolality is not clear. Nevertheless, this residual and relatively low AVP secretion should have only a weak action on the V2R mutants given their low affinity for AVP and, mainly, the reduced cAMP production level observed after stimulation by AVP. [11].

## 4. Diagnosis of NSIAD Patients

When NSIAD is suspected, the plasma sodium level, plasma and urine osmolalities, and plasma AVP level should all be measured at the same time during the hyponatremic episode. The association of hyponatremia with relatively high urine osmolality and a low or undetectable plasma AVP level is an indication for sequencing the *AVPR2.* In siblings, a water-load test can be performed in an asymptomatic boy but cautiously: for example, with only 10 mL/kg of water [5]. In girls, a 20 mL/kg water-load test can help to determine their ability to dilute their urine, as this is variable in heterozygous females due to random X inactivation and will help in providing them with recommendations for water intake.

Assessment of the concentration of the plasma von Willebrand factor (vWF) antigen, which is known to be increased by AVP stimulation of the so-called “extrarenal V2R,” does not seem to be helpful in diagnosing NSIAD patients [4].

## 5. Management of Patients with NSIAD

In symptomatic NSIAD patients with hyponatremia, rapid management with fluid restriction and, if necessary, administration of 30–50% oral urea solution, is necessary to avoid persistent or increased hyponatremia, which carries the risk of brain damage [9, 16].

In addition, hemizygous boys have to avoid massive water intake, as shown by the results observed during water-load testing. In these patients, caution is particularly important when environmental factors may encourage sharp rises in water intake (sports activities, during heat waves, in the summertime, etc.). However, boys bearing the mutation are mainly at risk of developing severe hyponatremia during infancy when their water intake is mostly under parental control. In heterozygous females, the advice will depend on the results of the water-load test and will range from normal to cautious water or fluid intake.

## 6. Conclusion

The prevalence of this recently recognized disease is difficult to determine in children, and adults. Although NSIAD does not seem to have an early neonatal expression, the diagnosis should systematically be considered in infants, children and adults presenting with hyponatremia associated with high urine osmolality. In males, the diagnosis will be easily made by sequencing the *AVPR2*. In siblings, a water-load test can be helpful in studying and advising female carriers. The treatment of symptomatic patients is based on fluid restriction and, if necessary, administration of an oral urea solution. Otherwise, treatment is mainly preventive and aims to avoid poorly adapted fluid intake.
